# Toxic Anterior Segment Syndrome after Foldable Artiflex Iris-Fixated Phakic Intraocular Lens Implantation

**DOI:** 10.1155/2011/982410

**Published:** 2011-06-05

**Authors:** Lucien A. M. van Philips

**Affiliations:** Afdeling Oogheelkunde, Medisch Centrum Haaglanden, Lijnbaan 32, 2512 VA Den Haag, The Netherlands

## Abstract

Toxic anterior segment syndrome (TASS) developed in four cases after uneventful implantation of a foldable iris-fixated phakic intraocular lens (pIOL). Two cases occurred sequentially in one patient. The TASS subsided without complications in all cases after intensive topical steroid treatment. A multitude of possible causes is considered for the occurrence of these TASS cases. From the sterilization and cleaning of surgical instruments to the possibility of endotoxines in ophthalmic viscosurgical devices (OVD). These rare cases should alert the surgeon to the possibility of TASS after pIOL implantation.

## 1. Introduction

Toxic anterior segment syndrome (TASS) is an acute, sterile anterior segment inflammation following any anterior segment surgical procedure [1]. Usually the anterior segment inflammation starts within 12–48 hours after surgery. Clinically alarming symptoms include diminished visual acuity, increased intraocular pressure, corneal edema, inflammation of the anterior chamber, fibrin, hypopyon, and a fixed pupil [2–4]. TASS results from a noninfectious toxic agent within the anterior chamber [5, 6]. The offending substances include denatured ophthalmic viscosurgical devices (OVDs), preservatives, talc from surgical gloves, topical ophthalmic ointment, inappropriately reconstituted intraocular preparations, altered pH and osmolarity of intraocular fluids, heat stable endotoxins, and detergents [5, 6]. Mild to moderate cases respond well to corticosteroids [3, 4], while severe cases might lead to corneal decompensation, glaucoma, a permanently dilated pupil, and cystoid macular edema [2, 3, 7, 8]. TASS is most commonly reported after cataract surgery and rarely after phakic intraocular lens (pIOL) implantation [1, 9, 10]. In the literature, three TASS cases have been reported after pIOL implantation [11, 12]. This report presents three cases of TASS, two of which occurred sequentially in one patient after foldable Artiflex iris-fixated pIOL implantation.

## 2. Case Reports

**Case 1**

A 45-year-old woman with high myopia consulted our clinic for a refractive surgical procedure. The patient was not contact lens intolerant. History revealed no allergy, uveitis, rheumatic disease, or herpetic keratitis. Corrected distance visual acuity (CDV) in the right and left eye was, respectively, 1.0 and 0.80 with a manifest refraction of, respectively, −12.25 and −15.0–0.50 × 135. Photopic (85 candelas/m^2^) low contrast (2.5%) visual acuity (LCVA) preoperatively was 0.40 and 0.32 in the right and left eye, respectively. Mesopic (0.7 candelas/m^2^) LCVA preoperatively was 0.25 and 0.20 in the right and left eye respectively. Mesopic pupil diameter was 7 mm in both eyes (Colvard, Oasis Medical). Higher-order aberration (HOA) value for a 6 mm pupil diameter was 0.44 *μ*m in the right eye. Measuring the HOA for the left eye was out of limit for the Zywave II aberrometer (Bausch & Lomb). Tonometry measured 16 and 15 mmHg, respectively, in the right and left eye. Preoperative central endothelial cell density (cECD) was 2894 and 2822 cells/mm^2^ in the right and left eye (SP 2000P, Topcon Inc.). The thinnest corneal thickness was 526 *μ*m and 525 *μ*m in the right and left eye (Orbscan IIz, Bausch & Lomb). Anterior chamber depth was 3.39 and 3.33 mm in the right and left eye. Slitlamp examination and fundoscopy were unremarkable. The procedure of choice was implantation of a foldable Artiflex iris-fixated pIOL (Ophtec Inc., Groningen, The Netherlands) in both eyes. At least two weeks before surgery, a YAG laser peripheral iridectomy was performed in both eyes.

Preoperatively, the patient was treated with 1 drop of pilocarpine nitrate 2% preservative-free (Chauvin Pharmaceuticals Ltd., UK), oxybuprocaine hydrochloride 0.4% preservative-free (Chauvin Pharmaceuticals Ltd., UK) and tetracaine hydrochloride 1% preservative-free eyedrops (Chauvin Pharmaceuticals Ltd., UK). Three doses (*∼*40 *μ*L per dose) of each eyedrop were instilled on the ocular surface 10 min apart starting 30 min before surgery. One hour before surgery she received diazepam 5 mgr po. The periocular skin and conjunctiva (cul-de-sac) were cleansed with povidone-iodine 10% (active iodine 1% (Fresenius Kabi, The Netherlands)) solution at least 3 minutes before surgery. Lint-free gloves were used under topical anesthesia. To maintain pupil constriction intraoperatively, acetylcholine chloride (Miochol, Thea Pharma) was used.

The right eye was the first to be operated. First two vertical paracenteses were performed located at 2 and 10 o'clock and directed to the enclavation site. Intracameral viscoelastic material (Provisc, Alcon Laboratories, Inc., Fort Worth, Tex, USA) was introduced. After a corneoscleral incision of 3.2 mm located at 12 o'clock, the foldable Artiflex lens was inserted with the use of a specially designed spatula that allows the surgeon to fold and insert the lens. Special curved forceps were used for the enclavation by holding the PMMA haptics at the base. After careful removal of the Provisc from the anterior chamber with a disposable irrigating cannula, balanced salt solution (BSS PLUS, Alcon Laboratories, Inc., Fort Worth, Texas, USA) was injected into the eye. Thereafter cefuroxime 1.0 mg (Zinacef (GlaxoSmith Kline)) was injected intracamerally to prevent endophthalmitis [13]. Suturing was not necessary since the incisions were checked watertight. There were no intraoperative complications. After the operation, prednisone 1% (Pred-Forte (Allergan)) and ketorolac tromethamine 0.5% (Acular Allergan) drops were used four times a day. Ofloxacin 0.3% (Trafloxal (Tramedico)) drops four times a day were given for one week. The patient received acetazolamide (Diamox (Goldshield Pharmaceuticals)) 250 mgr orally.

**Figure 1:**
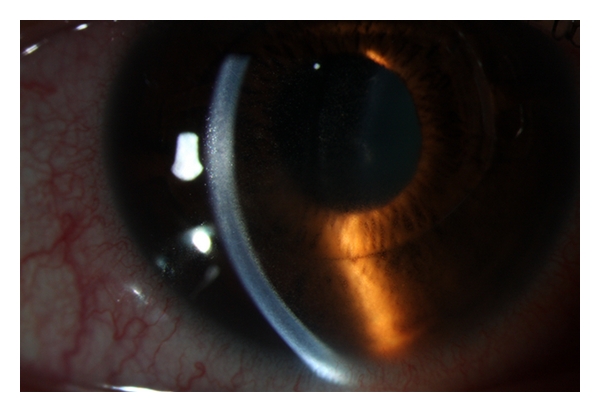
Toxic anterior segment syndrome (TASS) in the right eye, one day after implantation of a foldable Artiflex pIOL.

On the first postoperative day, the patient presented with a bruised feeling in the right eye and CDVA of 0.80. The patient had no pain. There was conjunctival and ciliary injection, without evident corneal edema. Slitlamp examination of the anterior chamber revealed diffuse descemet membrane keratic precipitates (KPs), 2+ immobile cells and flare with a round and sluggishly reactive pupil. There was some fibrin in the anterior chamber but no hypopyon (Figure 1) There were no cells in the vitreous. The intraocular pressure (IOP) was 18 mmHg. Topical prednisone 1% (Pred, Forte) one drop every hour, ultracortenol ointment (Novartis Pharma) for the night, and ketorolac tromethamine 0.5% (Acular) four times daily were prescribed. The patient was under close followup every day until after one week the anterior chamber reaction subsided, and the conjunctival injection was gone. There were no KPs or synechiae and the IOP was 13 mmHg. Fundoscopy was unremarkable. CDVA in the right eye was 1.2 with manifest refraction 0–0.50 × 102. Topical steroids were tapered. After an extensive discussion with the patient about the TASS syndrome, the various options and the risks of pIOL implantation in the fellow eye, a decision was made to implant an Artiflex pIOL without the application of cefuroxime intracamerally.

Two weeks after the first procedure an Artiflex pIOL was implanted in the left eye as described before, without the injection of cefuroxime intracamerally. There were no intraoperative complications.

**Figure 2:**
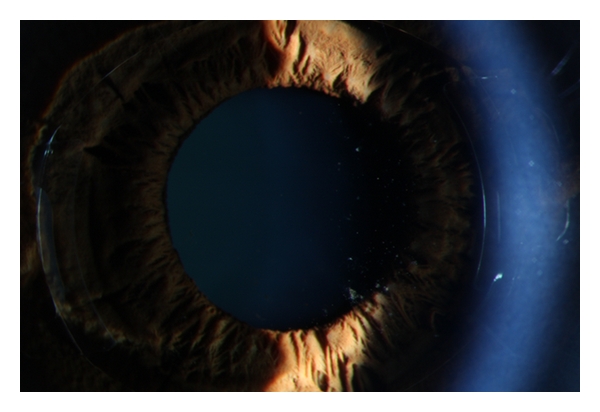
Cell deposits on the posterior surface of a foldable Artiflex phakic pIOL in the right eye, two months after surgery. The TASS has subsides.

On postoperative day 1, CDVA in the left eye was 0.80 with −1.0–0.50 × 130. The patient had photophobia without pain. Slitlamp examination revealed conjunctival and ciliary injection without corneal edema. There was an anterior chamber inflammation with 1+ immobile cells and flare, slight fibrin without hypopyon. The pupil was round with slightly reduced reaction. No cells were apparent in the vitreous, and fundoscopy was normal. The patient was prescribed prednisone 1% (Pred Forte) one drop every hour, ultracortenol ointment (Novartis Pharma) for the night, and ketorolac tromethamine 0.5% (Acular) four times daily until the anterior chamber reaction subsided. Six days postoperatively CDVA was 0.90 with −1.0–0.50 × 130 in the left eye. There were no cells in the anterior chamber, and there was some pigment on the pIOL. IOP was 19 mmHg. Slitlamp examination three months postoperatively showed some cell deposits on the posterior surface of both pIOLs (Figure 2).

HOA value was 0.45 and 0.27 *μ*m in the right and left eye, respectively. Six months postoperatively, CDVA was 1.2 with 0–0.25 × 100 and 1.2 with −1.25 0 in the right and left eye, respectively. Mesopic and photopic LCVA were both 0.40 and 0.70/0.50 in the right and left eye, respectively. cECD was 2688 cells/mm^2^ and 2909 cells/mm^2^ in the right and left eye, respectively. Evaluation of the video recording of both procedures revealed no abnormalities. The duration of surgery was approximately 10 and 7 minutes in the right and left eye, respectively.

Skin patch tests with cefuroxime, Provisc, and Miochol revealed no reactions. There was a 1+ (erythema with papules or induration) skin patch reaction with N-isopropyl-N′-phenyl-paraphenylenediamine (IPPD) (rubber antioxidant), nickel sulphate 5%, mercaptobenzothiazole 2% (rubber accelerator), sesquiterpene lactone mix 0.1%, and tixocortol-21-pivalate 1%.

**Case 2**

**Figure 3:**
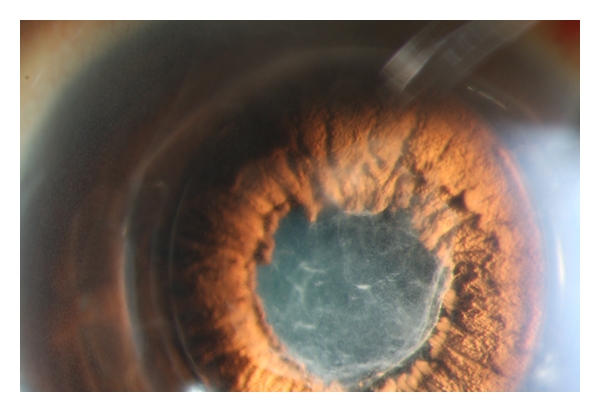
Severe fibrinous reaction one day after foldable toric pIOL implantation in the anterior chamber.

**Figure 4:**
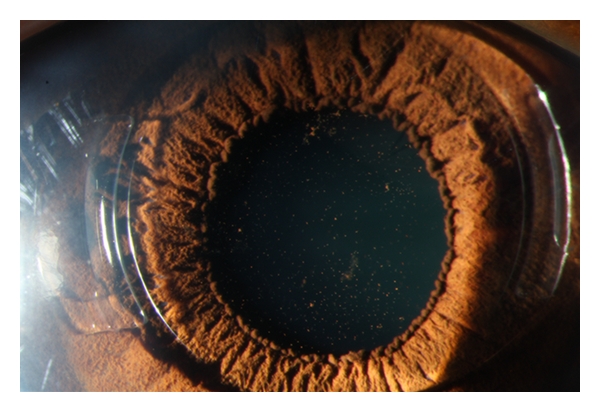
Pigment deposits on the posterior surface of a foldable toric Artiflex pIOL one week after surgery.

A 33-year-old woman received bilateral toric Artiflex pIOL implantation for the correction of her myopia and astigmatism. History was unremarkable. CDV in the right and left eye was, respectively, 0.70 and 0.90 with a manifest refraction of, respectively, −8.00–2.50 × 5 and −6.50–2.25 × 169. Photopic LCVA preoperatively was 0.25 and 0.30 in the right and left eye, respectively. Mesopic LCVA preoperatively was 0.25 and 0.30 in the right and left eye, respectively. HOA value for a 6 mm pupil diameter was 0.37 *μ*m in the right eye and 0.44 *μ*m in the left eye. Tonometry measured 12 and 10 mmHg, respectively, in the right and left eye. Preoperative cECD was 3184 and 2860 cells/mm^2^ in the right and left eye. Anterior chamber depth was 3.52 and 3.58 mm in the right and left eye. White-to-white was 11.6 mm in both eyes. Slitlamp examination and fundoscopy were unremarkable. The procedure of choice was implantation of a foldable toric Artiflex iris-fixated pIOL (Ophtec Inc., Groningen, The Netherlands) in both eyes. A pIOL of −8.50 D −2.0 × 0 and −7.00 D −2.0 × 0 was implanted in axis 5 and 169 of, respectively, the right and left eye. The preoperative preparation and pIOL implantation in both eyes was performed as described in Case 1. Both eyes received cefuroxime intracamerally. The right eye was operated first. One day postoperatively, the patient had blurred vision without pain. There was ciliary injection with no evident corneal edema. Slitlamp examination of the anterior chamber revealed diffuse descemet membrane KPs, 2+ immobile cells and flare with a round and sluggishly reactive pupil. There was extensive fibrin in the anterior chamber with a hypopyon (Figure 3). There were no cells in the vitreous. The intraocular pressure (IOP) was 9 mmHg. Intensive topical steroid treatment was administered until the anterior chamber reaction subsided. Four days postoperatively, there were no KPs or fibrin in the anterior chamber (Figure 4), and the IOP was 11 mmHg. Fundoscopy was unremarkable. CDVA was 1.0 with 0–0.50 × 110. Topical steroids were tapered. The left eye subsequently received a foldable toric Artiflex pIOL with a peroperative iris prolaps, which was repositioned. There were no postoperative complications. Six months postoperatively CDVA was 1.0 with 0.75-0.75 × 110 and 1.2 with 0–0.75 × 55 in the right and left eye, respectively.   Mesopic and photopic LCVA were both 0.32 and 0.40 in the right and left eye, respectively. HOA value for a 6 mm pupil diameter was 0.53 *μ*m in the right eye and 0.39 *μ*m in the left eye. cECD was 3088 cells/mm^2^ and 2916 cells/mm^2^ in the right and left eye, respectively.

## 3. Discussion

Toxic anterior segment syndrome (TASS) is a potentially devastating acute inflammatory reaction in the anterior chamber and is increasing in recognition [1, 11, 12, 14–16]. It is most commonly reported after cataract surgery, but has been also reported after pIOL implantation, penetrating keratoplasty and intravitreal injection with bevacizumab [1, 11, 12, 14, 16–18]. TASS should be differentiated from postoperative endophthalmitis. Clinically TASS is characterized by intense early postoperative inflammation of the anterior segment, fibrin formation, and corneal edema [1, 14, 16]. Patients with TASS usually do not have perocular pain, and there is rarely any vitreous inflammation. The TASS cases presented in this report had minimal decrease in CDVA with intense anterior chamber inflammation, fibrin formation, and hypopyon. There was no increased intraocular pressure, corneal edema, or vitritis. The cases occurred within 24 hours after iris-fixated pIOL implantation and responded well to intense steroidal treatment, which is typical for TASS, differentiating it from endophthalmitis. Hence taking culture samples from the aqueous humor or vitreous was not necessary. Moshirfar et al. reported a case of TASS after rigid iris-fixated pIOL (Verisyse) implantation with severe corneal edema, which resolved over a period of 2 months with steroidal treatment [12]. After one year corneal endothelial cell density had decreased by 69%. The authors attributed the endothelial cell loss to a toxic insult consistent with TASS. A definite toxic substance was not identified. Another study reported two cases with TASS, one after rigid (Verisyse, AMO, Abbott Park Ill, Usa) and one after foldable (Artiflex, Ophtec BV, The Netherlands) iris-fixated pIOL implantation, with severe fibrinous anterior chamber inflammation, hypopyon, and nonsignificant corneal edema [11]. Anterior chamber washout and aspiration of fibrin membranes were performed. Intracamerally ceftazidime and vancomycin were injected. Cefazolin and garamycin drops were instilled until anterior chamber fluid cultures were negative. After intensive steroidal treatment the TASS subsided in both cases within one week. The authors suggested that  Multivisc BD was the toxic agent causing TASS [11]. 

Numerous noninfectious substances have been implicated to cause TASS: abnormal pH and osmolarity, anesthetic agents, preservatives, intraocular lenses, irritants on the surfaces of surgical instruments (denatured OVDs), heat stable endotoxins, impurities of autoclave steam, and topical ophthalmic ointments [5, 6]. The multitude of possible causes of TASS makes it difficult to prevent. The TASS task force of the American Society of Cataract and Refractive Surgery (ASCRS) has developed a questionnaire to assist investigation of a TASS outbreak (http://www.ascrs.org/TASS/Instrument-Re-processing-Product-Questionnaire-Survey.cfm). Two of the three TASS cases presented in this study occurred in the same patient and sequentially. The three cases probably do not constitute an outbreak. There were no other cases of TASS at the surgical center in the same period. The surgical instruments used in both cases were either disposable (syringes, enclavation needle, and cannulas) or nondisposable (titanium spatula and forceps) and were rinsed and sterilized according to a strict protocol. Surgical instrument cleaning and sterilization cannot be ruled out as the cause of these TASS cases. After the first TASS case cefuroxime was considered a possible toxic or allergic agent, even though previous surgeries had no toxic outcome. Thus in the fellow eye cefuroxime was not used. To minimize the risk of dosage errors the intracameral cefuroxime, for the first eye, was prepared in the hospital pharmacy and not in the operating theatre. Though cefuroxime seems safe to use intracamerally, in patients with vulnerable corneal endothelial cells, it may be more toxic [19]. The sequential TASS in the fellow eye of Case 1 in this paper probably rules out cefuroxime as a cause. Miochol could not be ruled out as a cause, but no correlation with TASS has been reported. The pIOL design and compound (polymethyl methacrylate (PMMA) haptics and silicone optic) were also considered as potential causes of TASS. The lot numbers of both Artiflex lenses were different. It has been reported that after foldable Artiflex pIOL implantation giant cells with pseudopodia adhere to the posterior surface of the lens and that 4.8% develops pigment deposits on the pIOL [19, 20]. Whether these rare long-term complications indicate a predisposition to TASS development remains unclear. Two TASS cases in this report developed some cell deposits on the posterior surface of the pIOL. The third case had some pigment precipitates on the pIOL. Although possible, it is unlikely that povidone iodine entered the anterior chamber, since the concentration used in both eyes would have been highly toxic to the corneal endothelium and no corneal edema was noted [21, 22]. The Provisc used in these cases did not contain dry natural rubber, which is another potential cause of TASS [5, 6].

Viscosurgical devices can be produced by gene-coded bacteria in a microbial fermentation process and may be contaminated by heat stable endotoxins [11]. Endotoxins in OVDs must not exceed 0.50 endotoxin units/mL [23]. The highest acceptable endotoxin concentration (EC), however, is yet to be established. In one study, Provisc had an EC under 1.2 endotoxin units/mL [24]. Concern has been expressed regarding the presence of endotoxins in OVDs, which may be responsible for postoperative anterior chamber reactions [11, 25]. The use of pure OVDs is therefore recommended to prevent inflammatory reactions [24].

The unique sequential TASS development in both eyes of one patient, presented in this paper, suggests a unique reaction to a noninfectious substance. One hypothesis might be that heat-stable endotoxins in the Provisc caused an anterior segment inflammatory reaction in a hypersensitive patient. Implantation of a foldable iris-fixated pIOL carries a risk of viscosurgical remnants in the eye; therefore, thorough irrigation at the close of surgery is recommended. This paper serves to alert surgeons to the possibility of TASS after implantation of a foldable iris-fixated pIOL.
